# Schistosomiasis vaccines: where do we stand?

**DOI:** 10.1186/s13071-016-1799-4

**Published:** 2016-09-30

**Authors:** Biniam Mathewos Tebeje, Marina Harvie, Hong You, Alex Loukas, Donald P. McManus

**Affiliations:** 1 QIMR Berghofer Medical Research Institute, Brisbane, Australia; 2 School of Public Health, University of Queensland, Brisbane, Australia; 3 Department of Immunology and Molecular Biology, School of Biomedical and Laboratory Sciences, College of Medicine and Health Sciences, University of Gondar, Gondar, Ethiopia; 4 Centre for Biodiscovery and Molecular Development of Therapeutics, Australian Institute of Tropical Health and Medicine, James Cook University, Cairns, Australia

**Keywords:** *Schistosoma mansoni*, *Schistosoma haematobium*, *Schistosoma japonicum*, Immune response, Schistosomiasis, Vaccine, Immune protection, Antigen discovery

## Abstract

Schistosomiasis, caused mainly by *S. mansoni*, *S. haematobium* and *S. japonicum,* continues to be a serious tropical disease and public health problem resulting in an unacceptably high level of morbidity in countries where it is endemic. Praziquantel, the only drug currently available for treatment, is unable to kill developing schistosomes, it does not prevent re-infection and its continued extensive use may result in the future emergence of drug-resistant parasites. This scenario provides impetus for the development and deployment of anti-schistosome vaccines to be used as part of an integrated approach for the prevention, control and eventual elimination of schistosomiasis. This review considers the present status of candidate vaccines for schistosomiasis, and provides some insight on future vaccine discovery and design.

## Background

The World Health Organization (WHO) considers schistosomiasis to be second only to malaria as the most devastating parasitic disease in terms of socioeconomic importance and public health impact [1]. Human infection is due to three main species, namely *Schistosoma mansoni* and *S. japonicum* which cause intestinal/hepatic schistosomiasis [2, 3] and *S. haematobium,* which results in urinogenital disease [4].

Human treatment with praziquantel (PZQ) is playing a central role in the control and prevention of schistosomiasis, being the only effective drug currently available [5]. However, the drug does not prevent re-infection and its exclusive use for the prevention and control of schistosomiasis is problematic; having been used for more than three decades, the emergence of PZQ-resistant schistosomes is a constant threat. Other drawbacks with PZQ are its poor activity against immature schistosomes, resulting in sub-optimal outcomes during mass drug administration campaigns and, as its mechanism of action remains unclear, the design of alternative drug formulations has proven difficult [5]. Furthermore, a substantial infrastructure is required to ensure the drug is supplied regularly in timely fashion to all parts of an endemic area.

Originally used as a major preventative measure [6], snail control, through the use of molluscicides (e.g. niclosamide), is now not the recommended method in isolation for the prevention of schistosomiasis [5]. In order to control and finally eliminate schistosomiasis, a vaccine will likely be a key component of an integrated approach (i.e. involving mass chemotherapy, targeted mollusciciding, environmental modification, health education, improved sanitation and vaccination). A transmission blocking vaccine for use in bovines could serve as a vital component in the control of *S. japonicum* [7], whereas clinical vaccines against *S. mansoni* and *S. haematobium* need to be developed. However, it is a sobering thought that no commercial vaccine is available currently against any of the human schistosomes, thereby emphasising the need for continued efforts towards achieving this goal. This review evaluates the current status of schistosome vaccine development.

## Strategies for vaccine development

Although complex, the schistosome life-cycle, with its various stages each expressing distinct antigens, provides a vehicle for identifying many alternative molecules for vaccine development. The fact that the different stages reside in different host niches (larvae in the skin and lungs, adults in the liver and intestine or bladder capillaries) can help in the design of possible vaccines to prevent the migration of schistosome parasites and their maturation to adult worms. Importantly, the fact that schistosomes do not replicate in the definitive host makes partial reduction of the parasite burden sufficient to control schistosomiasis, strengthening the argument for developing an effective vaccine as a control intervention [8]. When identifying a suitable vaccine candidate, it is prudent to select key schistosome molecules in the live parasite that are (a) exposed to the host immune system; and (b) are essential for parasite survival. Such components may, for example, function in migration, immune evasion, nutrient uptake or attachment.

Adjuvant selection and mode of vaccine formulation and delivery are other important considerations in vaccine design and deployment as they can have a considerable impact on the protective effectiveness of the vaccine. It is well known that, in contrast to attenuated cercarial vaccines, other types, such as subunit vaccines, require an appropriate adjuvant to help stimulate the immune system. A number of different adjuvants are available such as gels, emulsions, particulates, cytokines, microbial products (e.g. CpG, cholera toxin) and proteases. Adjuvants can overcome immune senescence in older individuals, prolong the immunological memory of a vaccine, broaden the antibody repertoire and direct the immune system to a Th1 biased, Th2 biased or mixed Th1/Th2 biased response [9]. For example, a Th1 driving adjuvant such as IL-12, administered with irradiated cercariae, provided up to 90 % protection in murine schistosomiasis [10, 11]. Currently, in order to increase the protective efficacy of schistosome vaccines, a strategy of combining existing adjuvants with novel ones, developed based on emerging immunological targets, has been muted [11].

Some of the other challenges in schistosomiasis vaccine development are the risk of an atopic IgE response to a candidate vaccine [12], a lack in understanding of the nature of the immune response and the correlates of protective immunity in humans and other mammalian hosts, the transmission of other pathogens in schistosomiasis endemic areas resulting in co-infected individuals which can impact on vaccine efficacy, and antigenic polymorphism [13].

## *Schistosoma mansoni* and *S. haematobium* vaccine candidates

Over 100 schistosome vaccine antigens have been identified, of which about a quarter have shown some level of protection in the murine model of schistosomiasis [14]. Disappointingly, however, only three molecules, *S. mansoni* fatty acid binding protein (Sm14), *S. mansoni* tetraspanin (*Sm*-TSP-2) and *S. haematobium* glutathione S-transferase (Sh28GST), have entered human clinical trials with Smp80 (calpain) undergoing testing in non-human primates [15].

A recent report has suggested that the murine model of schistosomiasis may be intrinsically flawed for pre-clinical testing of vaccine candidates as a result of the fragility of the pulmonary capillaries in mice which can prevent maturation of a large proportion of schistosome cercariae upon challenge; this may lead to the incorrect assumption that vaccine antigen-induced acquired protective immunity has been generated [16]. This article has stimulated vigorous debate and its conclusions require rigorous testing but, with this caveat, some of the *S. mansoni* and *S. haematobium* vaccine candidates that have been identified are now highlighted below and in Table 1.

**Table 1:** Recent data on *Schistosoma mansoni* vaccine candidates

Antigen	Location in adult worm	Identity/Function	Immunization strategy	Adjuvant	Host	Worm burden reduction (%)	Liver egg burden reduction (%)	Reference
Sm-p80	Associated with tegument inner membrane	Calpain-neutral cysteine protease	Recombinant protein	Resiquimod	Mouse	50	16	[25–30]
			Primed with pcDNA3 and boosted with recombinant protein	Resiquimod	Mouse	49	30	
			Primed and boosted with recombinant protein	Oligodeoxynucleotide	Mouse	70	75	
			Recombinant protein	Resiquimod	Baboon	58	–	
			DNA vaccine	–	Baboon	38–46	32–28	
Fatty acid binding protein (FABP) (Sm14)	Whole body, Cytosolic	Absorbs, transports and compartmentalizes fatty acids from the host	Recombinant protein	–	Mouse	67	–	[18]
Tetraspanin protein 2 (Sm-TSP2)	Tegument apical membrane	Tetraspanin integral membrane protein	Recombinant protein	Freund’s	Mouse	57	64	[99, 100]
			Recombinant protein	Alum/CpG	Mouse	25	27	
Glutathione S-transferase (Sh28GST)	Whole body	Enzyme involved in fatty acid metabolism and prostaglandin D_2_ synthesis	Recombinant protein	Aluminium	Baboon	0–80	–	[101]
Sm29	Tegument apical membrane	Unknown, but has a C-terminal domain	DNA vaccine with pUMVC3 plasmid	–	Mouse	17–22	–	[44, 102]
			Recombinant protein	Complete Freund’s and Incomplete Freund’s	Mouse	51	–	
Sm14 + Sm-29	–	–	Multivalent recombinant proteins	Poly (I; C)	Mouse	40	68	[41]
Sm29 + Sm-TSP-2	–	–	Multivalent DNA vaccine with pUMVC3 plasmid	–	Mouse	24–32	–	[43, 44]
			Multivalent Recombinant proteins	CpG-Alum	Mouse	35	–	
Oesophageal gland secretion (Sm100.3)	Oesophagus	Digestive tract proteins (oesophageal)	Recombinant proteins	Freund’s	Mouse	25–32	33–44	[103]
Cathepsin B1(SmCB1)	Gut (gastrodermis)	Gut protease (cysteine peptidase)	Recombinant proteins	Postulated to have inbuilt adjuvant properties	Mouse	73	83	[104, 105]
(Combined with SG3PDH^a^ + PRX-MAP^b^)								
*S. mansoni* Cathepsin B (Sm-CB)	Gut (gastrodermis)	Gut protease (cysteine peptidase)	Recombinant proteins	CpG oligodeoxynucleotides	Mouse	59	56	
				Montanide ISA 750 VG	Mouse	60	62	
Schistosome cysteine proteinase, asparaginyl endopeptidase (SmAE) (Sm32)	Gut	Gut protease (Asparaginyl peptidase)	DNA vaccine	–	Mouse	No significant reduction	37	[106]
Lysosome-associated membrane glycoprotein (Sm-LAMP)	Gastrodermis	Processing of ingested blood	Recombinant protein	alum-CpG	Mouse	16–25	–	[49]
Dynein light chain proteins	Unknown	Evolutionarily conserved among different organisms	Recombinant protein	Alhydrogel	Mouse			[52]
- DLC 12						43		
- DLC 13						51		
*S. mansoni* Syntenin (SmSynt)	Intestinal tract	Scaffold supporting protein	Recombinant protein	Complete and incomplete Freund’s	Mouse	30–37	–	[107]
Radiation-attenuated cercariae	–	–	UV-attenuated	–	Mouse	43	73	[54]
Antioxidants								
- Cu/Zn cytosolic superoxide dismutase	–	–	DNA vaccine	–	Mouse	44–60	–	[46, 47]
- signal peptide-containing superoxide dismutase	–	–	DNA vaccine	–	Mouse	22–45		
- glutathione peroxidase enzymes								
	–	–	DNA vaccine	–	Mouse	23–55		
					Baboon	17.1		

^a^Glyceraldehyde 3-phosphate dehydrogenase

^b^peroxiredoxin

### Sm14

Schistosomes lack an oxygen-dependent pathway for the synthesis of sterols and fatty acids. Therefore, they are entirely dependent on the mammalian host to provide these essential lipids. Schistosomes use fatty acid binding proteins (FABPs) to absorb, transport and compartmentalize fatty acids from the host and, because of this critical biological function, Sm14 has long been considered a potential vaccine candidate [17]. Recombinant Sm14 (rSm14) provided up to 67 % protection in terms of reduced *S. mansoni* worm burden in outbred Swiss mice without the use of an adjuvant, and encouragingly, no autoimmune response was observed even though its structure is identical in basic form with mammalian host homologues [18]. It has been shown to be cross-species protective against both *S. mansoni* and *Fasciola hepatica* infection*.* Development of a dual vaccine effective against both fluke infections has great appeal in terms of human and animal health. Recombinant Sm14 with glucopyranosyl lipid adjuvant stable emulsion (GLA-SE) adjuvant entered and successfully completed a phase 1 clinical trial in healthy adult volunteers in Brazil, confirming its status as safe and immunogenic [19]. Further immunogenicity and safety phase 2 trials of rSm14 (adjuvanted with GLA-SE) are planned for schistosomiasis-endemic areas in Brazil and Africa [20].

### Sh28GST

Schistosome 28 kDa glutathione S-transferase enzymes play a role in fatty acid metabolism and prostaglandin D_2_ synthesis and may help the parasite evade the host immune system. The enzyme present in *S. mansoni* (Sm28GST) has been tested extensively as a recombinant protein vaccine in various experimental models and has shown partial protection in terms of reduced worm burdens, inhibition of female worm fecundity and a reduction in egg viability [21]. Along with Sm14, Sm28GST was one of the six *S. mansoni* antigens originally independently tested under the auspices of TDR/WHO [22]. The *S. haematobium* homologue, Sh28GST (Bilhvax), formulated with alum adjuvant, has undergone human clinical trials [19, 23]. Cytokine production triggered by this vaccine candidate was shown to be influenced by factors such as host age, schistosome infection status and PZQ treatment history and this has provided an indication as to the features which should be considered for determining the efficacy of the GST-based vaccine during its testing in targeted endemic communities [23]. The Phase 1 and 2 clinical testing showed Bilhvax was safe for healthy and infected adults and children [13]. It was scheduled to complete a phase 3 self-contained, randomized, double blind clinical trial in 2012 (https://clinicaltrials.gov/ct2/show/NCT00870649) evaluating whether co-administration of the vaccine with PZQ could delay pathology relapse due to *S. haematobium* infection in children. The trial results, however, have yet to be released which has raised doubts about the effectiveness of the vaccine.

### Sm-p80

Calpain is a calcium activated neutral cysteine protease [24]. Prime-boost vaccination (priming with DNA and boosting with recombinant protein) with Sm-p80, the large subunit of *S. mansoni* calpain, in combination with resiquimod adjuvant, resulted in 49 % worm burden reduction, while 50 % protection was achieved using the recombinant protein as primary and boost vaccine (immunized and then boosted by recombinant protein) in mice [25]. With the same approach, but using a different adjuvant, oligodeoxynucleotide (ODN) 10104, 70 % worm burden reduction and 75 % egg reduction was achieved with Sm-p80 [26]. Moreover, a 58 % worm burden reduction in baboons (*Papio anubis*) was reported recently with the Sm-p80-based vaccine adjuvanted with resiquimod and CpG ODN [27].

Using a different approach, a Sm-p80 DNA vaccine conferred 59 % worm burden reduction and 84 % decrease in egg production in mice [28]. In baboons the vaccine provided levels of protection against *S. mansoni* infection comparable to those achieved by the irradiated cercarial vaccine; moreover, antibodies and IFN-γ were shown to play an important role in the protective immunity generated in this non-human primate model [29, 30].

Importantly, recombinant Sm-p80 has also been shown to exhibit cross-species protection against *S. haematobium* in hamsters and baboons [31]. Promisingly, enduring antibody titers were detected in mice at sixty weeks post-vaccination with recombinant Sm-p80, and IgG specific for Sm-p80 was detected in baboons 5–8 years after initial vaccination with the Sm-80 DNA vaccine [32]. It is anticipated that the recombinant Sm-p80/GLA-SE vaccine, “SchistoShield”, will move forward to phase 1 and 2 human clinical trials in 2017 [31, 32]. Furthermore, it has also been shown that Sm-p80 has a therapeutic effect in vaccinated baboons through decreasing the numbers of established worms, reducing the retention of eggs in tissues, and decreasing the number of eggs excreted in faeces [33].

### *Sm*-TSP-2

The tetraspanins are a group of proteins that are highly abundant in the schistosome tegument where they are found at the outer-most membrane of the intra-mammalian stage of the parasite, and hence are highly exposed to the host immune system [34]. The major tetraspanin proteins in *S. mansoni* are *Sm*-TSP-1 and *Sm*-TSP-2 with the latter conferring protection in *S. mansoni* challenge animal models and also correlating with protective immunity in naturally resistant people [35]. *Sm*-TSP-2 is currently being developed by the Sabin Vaccine Institute Product Development Partnership as a 9 kDa recombinant *Sm*-TSP-2/Alhydrogel® vaccine in combination with the GLA-AF adjuvant; it has undergone toxicology studies [36], has shown good preclinical results, expression of the antigen can be readily scaled up [37] (http://www.sabin.org/updates/pressreleases/phase-1-clinical-trial-sm-tsp-2-schistosomiasis-vaccine), and the vaccine has completed a phase 1 clinical trial although the outcome is not yet known. Disappointedly, whereas high levels of IgG1 and IgG2 antibodies were generated in mice against the recombinant TSP-2 protein homologue from *S. japonicum,* no consistent protective efficacy was achieved [38].

### Sm29

Sm29 is present in the tegument of adult worms and schistosomula and in its recombinant form it induces high level production of IgG1 and IgG3 isotypes among individuals resistant to infection and re-infection [39]. There are reports that recombinant Sm29 can prevent infection in animals previously exposed to *S. mansoni*. For example, 26–48 % protection was observed in BALB/c mice that were previously infected with a Brazilian strain of *S. mansoni* and treated with PZQ [40]. Recently an increased level of protection was obtained through combining Sm29 with Sm14 in the presence of poly I:C adjuvant; 40.3, 68.2 and 57.9 % reductions in adult worm burden, liver egg burden and intestinal eggs, respectively, was achieved, along with a reduction in granuloma size and number in the livers of immunized mice [41]. Another report showed that by fusing Sm29 and Sm14, a 48.4 % reduction in adult worm burden in mice was achieved [42]. Similarly, fusing Sm29 with *Sm*-TSP-2 resulted in an increased reduction (from 22 to 35 %) in the number of worms, a higher titre of IgG1 and IgG2 antibodies and increased levels of IFNγ and TNFα than Sm29 alone in challenged mice [43, 44]. In a further advance, recombinant Sm29 was subjected to high hydrostatic pressure which dissociated the aggregated protein resulting in a successfully folded, soluble, stable and structured molecule produced in high yield, which was protective against *S. mansoni,* thereby paving the way for its industrial production down track [45].

### Antioxidants

The antioxidants Cu–Zn superoxide dismutase (SOD) and glutathione S peroxidase (GPX) induced greater than 40 % reduction in worm burdens when administered as DNA-based vaccines in *S. mansoni*-challenged mice [46]. Similarly, a recent study showed a protective effect against schistosomiasis in baboons [47]. These non-human primates, vaccinated with two different formulations of SOD (SmCT-SOD and SmSP-SOD) and one of GPX, with a protocol of priming with naked DNA and boosting with the respective recombinant antioxidant proteins encapsulated in polylactic acid (PLA) microspheres, exhibited a robust immune response, which resulted in a reduction in worm numbers, and a pronounced anti-pathology effect compared with control animals [47].

### Digestive tract proteins

*Schistosoma mansoni* worms ingest host blood which passes through the oesophagus before arriving at an area in the gut where many peptidases catalyse its proteolysis. The processing of the blood and the resulting uptake of nutrients are functions essential for the survival of the parasite. Blocking these critical processes represents an important strategy for vaccine development and a number of digestive tract proteins, that are not recognized by host immune responses during normal infection, but are essential for parasite survival, have been tested as cryptic vaccine candidates [48]. One example was the trialling of a soluble form of schistosome lysosome-associated membrane glycoprotein (Sm-LAMP), located in the gastrodermis, which resulted in a reduction in worm burden (16–25 %) and in faecal eggs (52–60 %); moreover, its insoluble form produced up to 38 % reduction in liver egg burden [49]. Another recent study identified a number of esophageal secreted proteins, encoded by microexon genes (MEGs), that are involved in the initial processing of ingested blood and these, along with lysosomal hydrolase, also localised to the oesophagus, may prove to be novel immune targets [50]. Other examples of digestive proteins as potential vaccine targets are described in Table 1 and elsewhere [51].

### DLC/LC8

Proteins with the dynein light chain family (DLC/LC8) domain, which is evolutionarily conserved in different organisms, have been considered recently as vaccine targets against schistosomiasis as some homologues, such as the TAL family (Tegumental Allergen Like), are present in the distal cytoplasm of cells, in the tegument and on the membranous surface of schistosomes [52]. Recombinant *S. mansoni* DLC 12 and DLC 13, adjuvanted with alhydrogel, demonstrated a 43 and 51 % reduction in worm burdens respectively, in vaccinated and challenged mice [52]. Furthermore, both DLCs reduced the size of granulomas in hepatic tissues by up to 70 %. Their encouraging immunogenicity and protective efficacy, coupled with the absence of any allergic reactivity, warrants their further study as individual vaccine antigens or as part of a multi-component vaccine [52]. As DLC proteins are associated with motor myofibrillar proteins with intracellular function, the mechanism by which DLC immunization conferred protection remains to be determined.

### Attenuated *S. mansoni* vaccines

*Schistosoma mansoni* cercariae, attenuated by heat, chemical or ultraviolet treatment, or by ionizing radiation (gamma or X-ray), have been shown to provide protection against *S. mansoni* challenge in several mammalian species [24, 53]. Recent studies undertaken with UV-radiation-attenuated cercariae given once or, more effectively, multiple times to C57BL/6 mice have confirmed earlier findings with significant reductions in worm and hepatic and intestinal egg numbers in vaccinated animals [54, 55]. Moreover, tegumental changes in the adult worms (swelling, fusion of tegumental folds, vesicle formation and loss or shortening of spines on the tubercles) were evident [54, 55].

A recent systematic meta-analysis and review of the available publications in the area on mice indicated the irradiated cercarial vaccine has the potential to achieve protection as high as 78 % with one vaccination dose [53]. Although the models showed the generated protection waned, it remained elevated for at least eight months after vaccination, reinforcing the view that the level of protective immunity obtained, although partial, would reduce both schistosome transmission and parasite-associated morbidity [53]. This re-emphasises the potential of developing an attenuated cercarial vaccine, a concept proposed some years back that might well be worth revisiting [56], with the caveat that it would in all likelihood be problematical for human use (perhaps less so for application in reservoir hosts of *S. japonicum*), since such an attenuated vaccine would, for example, likely carry too high a risk of side effects or of partially or unattenuated parasites reaching the mesenteric veins and becoming patent. Long-term storage of such a vaccine before deployment is another challenge that would need to be overcome.

## Vaccine candidates for *S. japonicum*

Whereas clinical vaccines will need to be developed against *S. haematobium* and *S. mansoni,* the zoonotic nature of schistosomiasis japonica allows for a complementary approach for *S. japonicum* involving a transmission blocking vaccine for livestock animals, particularly bovines [24]. The vaccine would be used in reservoir hosts of *S. japonicum* to reduce transmission to humans [24].

### Attenuated *S. japonicum* vaccines

Vaccination of bovines with gamma irradiated schistosomula resulted in a significant reduction in adult worms and liver eggs compared with control animals [57]. Moreover, vaccination of pigs using UV-attenuated cercariae, produced variable, although highly promising, levels of protection in terms of reduced adult worms and hepatic egg burden in animals receiving a single immunization or those vaccinated three times [58]. The protective response was shown to be associated with IFNγ and IgG2 antibody production [59]. A pilot study in miniature pigs, using similar methodology (but incorporating a lower number of challenging cercariae), resulted in more than 80 % worm reduction [60]. However, it has been reported that unstable and relatively low protection is induced in C57BL/6 mice by attenuated *S. japonicum* cercariae. This is likely due to the poor Th1 response generated and the less robust antibody response produced in comparison with BALB/c mice, and suggests this mouse strain might be a sub-optimal model for studying the mechanisms of immune protection against this schistosome species [61].

### Sj97

Paramyosin is a myofibrillar 97 kDa protein present in the muscle layers and the tegument of schistosomes that has long been regarded as a vaccine candidate against *S. japonicum* and *S. mansoni* infection [24]. An early study showed that mice vaccinated intraperitoneally with purified paramyosin (without the use of an adjuvant) stimulated 62–86 % resistance against *S. japonicum* cercarial challenge [62]. In addition to preventing infection, a longitudinal treatment-re-infection design study in Leyte, the Philippines showed that a Th2 bias in response to Sj97 predicted a longer time to human re-infection and a lower re-infection intensity after treatment with PZQ [63, 64]. Moreover, it was reported in the human Leyte cohort that individuals who produce IgE but not IgG4 in response to rSj97 had 77 % lower re-infection intensity after 12 months >of treatment with PZQ [64, 65]. Sj97 is now in early preclinical testing with process development and further proof of concept studies taking place in mice and water buffalo [13, 64].

### Sj26GST

A *S. japonicum* 26 kDa GST plasmid DNA vaccine (Sj26GST) resulted in a significant reduction in worm numbers and in hepatic and faecal eggs in vaccinated mice; when the DNA vaccine was given in combination with interleukin 18 (IL-18), a potent IFN-γ inducing factor, the protective efficacy was improved [66]. DNA vaccines have some advantages over other types of immunization but they have some limitations concerning the gene delivery system. A recent study reported on a novel nanoparticle formulation of the Sj26GST DNA vaccine; although there was no significant reduction in worm burden, a highly significant decline in tissue egg burden and the fecundity of female adult worms resulted [67]. Toll-like receptor (TLR) 7/8 ligands (e.g. R848) and TLR 9 ligands (e.g. CpG oligodeoxynucleotides, or CpG) as adjuvants can increase vaccine effectiveness through activating the innate immune system and ultimately activating and directing the adaptive immune system. Such adjuvants have been shown to potentiate the activity of the Sj26GST DNA vaccine in mice by increasing splenocyte proliferation, elevating IgG, IgG2a, IFNγ and TNFα levels, and preventing Treg-mediated immunosuppression [68]. In another development, Sj26GST alone, or in combination with fatty acid binding protein (SjFABP), expressed in recombinant pseudorabies virus (PRV) Bartha-K61, induced significant levels of specific immunity and protection in mice and, importantly, sheep, emphasising the potential effectiveness of this live vector for vaccination against schistosomiasis japonica in animal reservoirs [69].

### SjTPI

The glycolytic pathway enzyme triose-phosphate isomerase (TPI), found in all stages of the schistosome life-cycle, is another targeted vaccine candidate for schistosomiasis japonica. An early study showed that a *S. japonicum* (Chinese strain) TPI (SjCTPI) plasmid DNA vaccine (with or without an IL-12 DNA plasmid) protected pigs against challenge infection [70]. Synergistic enhancement of immunogenicity and protection in mice against *S. japonicum* challenge was achieved with codon optimization and electroporation delivery of the SjTPI DNA vaccine [71], showing a similar level of protection as a replication-defective recombinant optimized SjTPI (rAdV-SjTPI) adenoviral vaccine [72] which was enhanced using a heterologous prime-boost strategy [73]. A study, conducted in Chinese water buffalo with a DNA vaccine encoding SjCTPI alone or fused with bovine HSP-70 with booster immunizations co-administered using a plasmid encoding IL-12, resulted in a significant reduction in worm numbers, liver and faecal eggs and in faecal miracidial hatching [74]. SjCTPI, delivered by a heterologous “prime-boost” regimen, has been used to vaccinate bovines in China as part of a multi-component integrated control package [75].

### SjIRs

*Schistosoma japonicum* possess two types of insulin receptors (SjIRs) which, on binding to mammalian host insulin, can activate the parasite’s insulin pathway, which is pivotal for glucose uptake, growth, and maturation [76]. Recombinant ligands of both *S. japonicum* insulin receptor 1 and 2 (SjLD1, SjLD2), tested in vaccine/challenge trials in mice resulted in significant reductions in faecal egg output, in reduced mature intestinal eggs and stunting of adult worms [77, 78]. The retardation in growth of the worms likely resulted from reduced glucose uptake [77]. Furthermore, knockdown of the SjIRs using RNA interference (RNAi) resulted in their reduced expression coupled with a reduction in the transcription level of downstream genes within the insulin pathway that are associated with glucose metabolism and schistosome fecundity [79], thereby reinforcing their vaccine potential.

### Sj14

In early studies, Sj14 (fatty acid binding protein; SjFABP), the *S. japonicum* homologue of Sm14, generated no or only a limited level of protection [24], but when given to mice as a DNA vaccine with a plasmid coding for IL-18 as adjuvant, the level of protection was increased substantially [80]. The latter study also showed that SjFABP + IL-18 increased the Th1 immune response by producing a higher level of IFNγ and a lower level of IL-4 compared with mice vaccinated only with SjFABP [80]. Somewhat disappointedly, the Sj14 DNA vaccine, coupled with Sj26GST to form a bivalent DNA-based vaccine, resulted in a reduced level of protective efficacy [81].

### Sj23

Sj23, like *Sm*-TSP-2, a member of the tetraspanin family, is a 23-kDa surface-exposed integral membrane protein expressed in all infective parasite stages. It was shown in BALB/c mice to elicit a rapid humoral immune response dominated by IgG2a antibodies, but not IgG1, and did not provide protection against cercarial challenge after priming with recombinant Semliki forest virus (SFV) particles followed by a boost with recombinant protein [82]. A subsequent report of mice vaccinated with purified recombinant protein LHD-Sj23-GST (large hydrophilic domain of Sj23 fused with Sj26GST) in combination with one of three adjuvants (Freund’s adjuvant (FA), Montanide ISA 206 or Montanide ISA 70 M), and parasite challenged, resulted in high-level production of LHD-Sj23-GST-specific IgG1, IgG2a and IgG3 antibodies and significant reductions in worm burden [83]. In order to further improve on the level of protection, a multivalent DNA vaccine comprising Sj23, glyceraldedyde-3 phosphate dehydrogenase (SjGAPDH), SjFABP and Sj26 was tested in mice which resulted in very high levels of protective efficacy in terms of reduced worms (70.8 % reduction) and liver eggs (60.7 % reduction) [84]. Another study in mice, using three cocktail DNA vaccines encoding Sj23, SjCTPI and NP30, boosted by electroporation in vivo and a protein vaccine boost to this regimen, resulted in a 60 % reduction in adult worm numbers and more than 60 % reduction in the liver egg burden [85].

Details of these and a number of other vaccine candidates tested against *S. japonicum* challenge infection are presented in Table 2 and elsewhere [78].

**Table 2:** Recent data on *Schistosoma japonicum* vaccine candidates

Antigen	Location in adult worm	Identity/Function	Immunization strategy	Adjuvant	Host	Worm burden reduction (%)	Liver egg burden reduction (%)	Reference
Paramyosin (sj97)	Schistosomulum surface, tegument and acetabular glands	Binds complement and Fc region of IgG; proposed role in host immune evasion	Recombinant proteins	Alum or TiterMax	Pig	33–34	–	[108]
Sj26GST	Parenchymal region of male worm and in the parenchymal cells between the vitelline glands in the female worm	Catalyses detoxification of lipophilic molecules by thioconjugation	DNA vaccine	–	Mouse	30	45	[66, 69, 109]
26-kDaGST + IL-18	–	–	DNA vaccine	–	Mouse	49	51	
SjGP-3 **(**Sj26GST + PmyF3 (fragment of paramyosin))	–	–	Polyvalent subunit	CFA	Mouse	41–38	26–29	
rPRV/Sj26-KDaGST+ SjFABP	–	–	Recombinant pseudorabies virus	–	Mouse	39	45	
					Sheep	48	51	
Triose phosphate isomerise (SjTPI)	–	Glycolytic pathway enzyme						[71–74]
- with codon optimiized version			DNA vaccine	–	Mouse	50	57	
- with SjTPI + heat-shock protein 70			DNA vaccine	–	Water buffalo	52	61	
- with recombinant replication-defective adenoviral vectors			Replication defective adenoviral vector-based	–	Mouse	54	52	
- with prime-boost strategy			Adenoviral vectored prime and recombinant protein boost	Complete Freunds and Incomplete Freunds	Mouse	72	72	
Thyroid hormone receptor beta (SjTHRβ)	–	Interacts with thyroid hormone to modulate growth, development and differentiation, and metabolic processes	Recombinant protein	Montanide ISA 206	Mouse	27	29	[110]
*S. japonicum* fatty acid binding protein (SjFABP)	Whole body, cytosolic	Uptake, transports and compartmentalizes the fatty acids of the host	DNA vaccine	–				[80, 81]
- with IL-18					Mouse	38	45	
- with Sj26GST					Mouse	32	25	
Twenty-three kilo dalton integral membrane protein (Sj23) with different adjuvants	Surface-exposed	Facilitates parasite immune regulation	Recombinant protein	Freunds	Mouse	59	–	[83]
				ISA206	Mouse	26	–	
				ISA70M	Mouse	54	–	
Tetravalent DNA vaccine (SjFABP. Sj23/Sj26.SjGAPDH)	–	–	DNA vaccine	–	Mouse	71	61	[84]
Cocktail DNA with EP and protein vaccines (Sj23+ SjCTPI+ CDR3)	–	–	DNA vaccine boosted with recombinant protein	Complete Freunds	Mouse	59	67	[85]
Protein disulfide (SjPDI) isomerase	Tegument as well as other specialized excretory/secretory (ES)organs	Enzyme involved in disulfide bond formation and rearrangement and it interfere with the host immune	Recombinant protein	Montanide ISA 206 VG	Mouse	38	33	[111]
		Responses						
*S. japonicum* inhibitor apoptosis protein using adenovirus as live vaccine vector (Ad-SjIAP)		Involved in differentiation and development	With recombinant adenoviral vector	–	Mouse	38	32	[112]
Aldose reductase (SjAR)	Gynecophoral canal of adult male worms	Involved in antioxidant defence system	Recombinant protein	Freunds	Mouse	33	28	[113]
*S. japonicum* Sj-F1 (using *S. gordonii* as live vector) (Sj-F1)	Gene discovered when adult *S. japonicum* worm cDNA library was screened with by serum prepared against female *S. japonicum* antigens	Unknown	With *S. gordonii* as live vector	–	Mouse	21	35	[114]

## New antigen discovery: a way forward

New antigen discovery has been aided by major recent advances in schistosome genomics, transcriptomics and post-genomic technologies [86]. Proteomics is another important and now widely used tool that can identify potential vaccine targets with a focus on the protein constituents of different schistosome sources such as the host-parasite interface comprising tegument or gut [87]. Studies on the tegument have used a number of procedures including biotin-labelling of live parasites and subsequent isolation and characterisation of the biotinylated proteins using tandem mass spectrometry (MS/MS) to identify surface-located proteins, and therefore those accessible to host antibodies. Proteomics of the schistosome gut and its contents has shed new light on the functionality of this important region of the parasite [87]. Coupled with other approaches such as metabolomics, interrogation of the schistosome proteome, particularly the surface, provides a mechanism to identify important clinically-relevant proteins and those having potential as new vaccine targets [87].

Vaccinomics is another powerful innovation which provides a foundation for searching critical determinants of immunity and can promote antigen discovery and the design of novel vaccines for complex pathogens such as the schistosomes [88]. A recent vaccinomics approach for discovering novel schistosome antigens that may not be revealed by conventional proteomics involved the design and manufacture of an immunomics protein microarray, the first to be generated for a multi-cellular pathogen. Mostly surface-derived proteins (215 in total) from *S. japonicum* and *S. mansoni* were selected and they were produced using a rapid in vitro translation system, and then printed as a vaccine discovery tool [87, 89, 90]. The reactivity of microarray proteins can be measured with antisera from human patients or schistosomiasis-resistant/exposed animals using a labelled secondary antibody and a laser microarray scanner; highly reactive proteins are then assessed as putative vaccines.

One application of the array used antibodies from acutely- or chronically-infected Chinese individuals with early/advanced schistosomiasis japonica, and subjects exposed, but stool negative for *S. japonicum* eggs, for screening. This resulted in the identification of 25 immunodominant antigens, including a number of vaccine candidates, transporters, tetraspanin-related proteins, and unannotated proteins [91]. The array has also been screened for IgG subclass and IgE responses, using sera from a human Brazilian cohort of putatively resistant (PR) and chronically *S. mansoni*-infected (CI) individuals stratified by worm intensity levels (high, medium, low), determined by faecal egg counts, so as to identify antibody signatures reflective of protective *vs* non-protective immune responses [92]. Probing for IgE responses allowed the identification of antigens that might induce potentially deleterious hypersensitivity reactions if used as subunit vaccines in endemic populations so it was encouraging that the PR individuals did not mount an intense IgE response to these antigens compared with CI subjects [92].

This immunomics-based approach to schistosomiasis vaccine antigen discovery was further validated by the identification of targets of the protective IgG1 immune response in PZQ-induced resistant subjects exposed to *S. haematobium*; uncharacterized proteins and a number of recognised vaccine antigens (e.g. glucose transporters, tetraspanins, glutathione-S-transferases, calpain) were identified [93]. The same report described the use of sera from rhesus macaques experimentally rendered resistant to *S. japonicum* infection to screen for antigen targets, and the discovery of new and known vaccine candidates, including many recognized by the human subjects.

Another important application has been the immune screening of the schistosome microarray with antibody secreting cell (ASC)-probes [89, 90, 94], generated from lymph nodes draining the sites of larval *S. japonicum* migration [95]. This technique is especially advantageous for recognizing antigens with low immunogenicity (selective pressure may have an influence on important protective epitopes which evolve over time with low immunogenicity) or those only temporarily exposed to the immune response [90]. In one study, ASC probes (from skin and lung) and sera from semi-permissive rats and sera from susceptible mice were used to screen the array after infection and re-infection with *S. japonicum* [94]. A total of 29 antigens, including a number of recognised vaccine candidates and several *S. japonicum* homologues of human schistosomiasis resistance markers - the tegument allergen-like proteins - were differentially recognized by infected hosts from which eight proteins were prioritized as putative novel schistosome vaccine and diagnostic antigens [94]*.* In a related study, the protein microarray, screened with ASC probes generated from *S. japonicum*-infected rats, resulted in the identification of a novel antigen, termed *S. japonicum* Ly-6-like protein 1 (Sj-L6L-1) which shares structural and sequence features with the Ly-6 protein family and has several other features suggesting it is a promising vaccine candidate against the developing larvae [96].

## Conclusion

Schistosomiasis remains a substantial public health problem due to the very high levels of morbidity it causes in many parts of the world. Currently, treatment is entirely dependent on PZQ chemotherapy. As exclusive use of the drug may lead to the emergence of drug resistant strains, development and deployment of a vaccine as part of an integrated approach for prevention and control of schistosomiasisis is to be encouraged. Much of our current understanding of immunity and immune mechanisms against schistosomiasis rely on studies conducted on mice, but vaccines based on studies performed only in the mouse model could have undesirable effects if taken prematurely to human clinical trials. The recent concern raised about using mice for determining the efficacy of vaccine candidates further reinforces the argument that additional critical examination of any identified candidate vaccine antigen, whether or not it has foundation in acquired immunity is essential, and that moving to studies using larger models such as rabbits, pigs or bovines in the case of *S. japonicum,* or non-human primates for *S. mansoni* and *S. haematobium,* is clearly necessary. Furthermore, much of our knowledge regarding immune protection has resulted from studies focused on *S. mansoni*, so further studies on *S. japonicum* and, particularly*, S. haematobium* are needed. Similarly, protection levels of many candidate vaccines show improvement after modification of antigen formulation and improved delivery systems. Combining different genes or antigens can also result in higher levels of vaccine-induced protection. Targeting key biological functions of schistosomes such as tegumental integrity, fecundity, and nutrient uptake using RNAi represent key potential sites to target the parasites for elimination through vaccination [97]. Although in its infancy, CRISPR technology may provide a novel approach identifying specific protein-encoding schistosome genes for vaccine candidate discovery [98].

Schistosomiasis vaccine development has proven highly challenging and costly and new funding is required to promote the generation of a schistosome vaccine antigen pipeline, similar to that in place for many other infectious diseases, and to progress existing promising candidates into clinical trials. It is becoming apparent that mass drug administration alone will not eliminate schistosomiasis and that a vaccine will be an essential component of any future schistosomiasis control intervention toolbox.

## Abbreviations

ASC: Antibody secreting cell
CI: Chronically-infected
CPG: 5′-C-phosphate-G-3′
CRISPR: Clustered regularly interspaced short palindromic repeats
DLC: Dynein light chain
FABPs: Fatty acid binding proteins
GLA-AF: Glucopyranosyl lipid A
GLA-SE: Glucopyranosyl lipid adjuvant stable emulsion
LHD-Sj23-GST: (large hydrophilic domain of Sj23 fused with Sj26GST)
MEGs: Microexon genes
MS: Mass spectrometry
NP30: Murine monoclonal anti-idiotypic antibody NP30
ODN: Oligodeoxynucleotide
PLA: Polylactic acid
PR: Putatively resistant
PRV: Pseudorabies virus
PZQ: Praziquantel
R848: Resiquimod
rAdV-SjTPI: Replication-defective recombinant optimized SjTPI adenoviral vaccine
RNAi: RNA interference
rSj97: Recombinant *Schistosoma japonicum* paramyosin
rSm14: Recombinant Sm14
SFV: Semliki forest virus
Sh28GST: *Schistosoma haematobium* 28 kDa (kilodalton) glutathione S-transferase
Sj14: SjFABP, *Schistosoma japonicum* fatty acid binding protein
Sj23: *Schistosoma japonicum* 23-kDa surface-exposed integral membrane protein
Sj26GST: *Schistosoma japonicum* 26 kDa glutathione S-transferase
Sj97: *Schistosoma japonicum* paramyosin
SjCTPI: *S. japonicum* (Chinese strain) TPI
SjFABP: *Schistosoma japonicum* fatty acid binding protein
SjGAPDH: *Schistosoma japonicum* glyceraldedyde-3 phosphate dehydrogenase
SjIRs: *Schistosoma japonicum* insulin receptors
Sj-L6L-1: *Schistosoma japonicum* Ly-6-like protein 1
SjLD1, SjLD2: ligands of *Schistosoma japonicum* insulin receptor 1 and 2
Sm14: *Schistosoma mansoni* fatty acid binding protein
Sm28GST: *Schistosoma mansoni* 28 kDa glutathione S-transferase
Sm29: *Schistosoma mansoni* 29 kDa antigen
SmCT-SOD: Cu/Zn cytosolic superoxide dismutase signal peptide-containing SOD
Sm-LAMP: *Schistosoma mansoni* lysosome-associated membrane glycoprotein
Smp80: *Schistosoma mansoni* calpain
SmSP-SOD: Signal peptide-containing SOD
*Sm*-TSP-1: *Schistosoma mansoni* tetraspanin-1
*Sm*-TSP-2: *Schistosoma mansoni* tetraspanin 2
SOD: Superoxide dismutase
TAL: Tegumental Allergen Like
TDR: The Special Programme for Research and Training in Tropical Diseases
TLR: Toll-like receptor
TPI: Triose-phosphate isomerase
WHO: World Health Organisation
